# Testing the Structural Validity of the Self-Stigma Scale in Relatives of People with Autism in the Spanish Context

**DOI:** 10.3390/ijerph18147384

**Published:** 2021-07-10

**Authors:** José M. Aguilar-Parra, Maria M. Megias, Rubén Trigueros, Isabel Mercader, Carolina Fernández-Jiménez, Juan M. Fernández-Campoy

**Affiliations:** 1 Hum-878 Research Team, Health Research Centre, Department of Psychology, University of Almería, 04120 Almería, Spain; jmaguilar@ual.es (J.M.A.-P.); marmegias@hotmail.com (M.M.M.); jfc105@ual.es (J.M.F.-C.); 2 Department of Psychology, University of Granada, 18010 Granada, Spain; carolina@ugr.es

**Keywords:** self-stigma, family, autism, psychometric properties

## Abstract

Sometimes, relatives of children with autism experience feelings of guilt, abandonment and devaluation, as they tend to see themselves as the cause of their children’s illness. This causes social isolation and psychological discomfort. However, there are no scales that assess self-stigma. Therefore, the aim of the study is to show evidence of the validity and reliability of the Self-Stigma Scale in Relatives of People with Mental Illness in the Spanish context in relation to autism. A total of 287 progenitors participated in the study. For the validation and adaptation process, three confirmatory factor analyses, analysis of invariance across gender, reliability analysis and temporal stability, were carried out. The results revealed that the factor structure of the questionnaire was adequate, showing adequate levels of reliability.

## 1. Introduction

Autism is a neuropsychological developmental disorder that causes qualitative impairments in interaction, communication and social imagination, and signs of cognitive and behavioural inflexibility, which functionally limit individuals and are present in a wide range of conditions [1,2]. The diagnosis of this disorder usually occurs during childhood, causing trauma for parents, often leading to a range of negative feelings (e.g., fear, worry, guilt, etc.) and psychological repercussions, such as stigma of association (or self-stigma), which consists as an extension of the concept by suffering rejection and marginalisation [3,4]. However, to date, researchers have not paid much attention to the repercussions of children’s autism on parents [5]. Therefore, the aim of the present study will be to test the factorial structure of the scale (Appendix A) to assess the self-stigma of relatives of people with autism.

Stigma is a concept that has been studied from two components: public stigma and self-stigma. The former refers to the social attitudes of the population towards a collection of people who are part of a certain group [6]. Self-stigma, on the other hand, refers to the internalisation of a negative social view, i.e., the process of internal acceptance of stigmatisation by people belonging to a particular group and their families (see Pescosolido and Martin [6]). This social perception causes people to make a series of internal attributions when seeking help from professionals, fearing that they will be socially labelled as emotionally unstable, less interesting and less self-confident than other individuals [6,7]. The main source of stigma and self-stigma among family members of people with autism is found in personal attributions emanating from popular culture about the etiology of the disorder [7]. Confusion about the diagnosis can also lead to difficulties to the point of isolation by other family members and friends, marital relationship problems, and parents may blame themselves for the child’s autism [8]. In addition, there is a social belief that the ways of upbringing [9], the genetic load [10,11] and the inoculation of vaccines are responsible for the development of the disorder in children [12]. These factors are coupled with the fact that families face a number of difficulties such as trying to understand their child’s behaviour and confusion about the nature of these difficulties [13]. 

Moreover, the media and communication media sometimes promote a biased, sensationalist and distorted image of this disorder, contributing to perpetuation and maintenance of unrealistic beliefs about the origin of autism. Besides, the lack of information, or the vague and imprecise knowledge that relatives sometimes have about the origin of the disorder, places them in a position of vulnerability, understanding these data as true and increasing the blame they place upon themselves [14,15]. In this way, relatives assume a role as an etiological agent, with feelings and behaviours linked to guilt, anxiety, abandonment and isolation appearing [16,17]. In this way, family members may have difficulties or avoid establishing interpersonal relationships, anticipating rejection and discrimination, and their self-esteem and general state of mind are also diminished [18].

At present, there are no scales known that evaluate the self-stigma of relatives of children with autism. In spite of this, from the mental health field, there is only one scale that assesses this state among relatives of people with several mental disorder (SMD), being the Self-Stigma in Relatives of People with Mental Illness (SSRMI; [19]). This scale is made up of a total of 30 items distributed among five subscales (stereotyping, collectively held undesirable characteristics, which are assumed to be shared by people in a stigmatised group, and are endorsed and internalised by family members; separation, family members view themselves and their loved ones as distinctly different from other people; discrimination, family members’ feelings of having been moved in a downward direction on the status hierarchy, leading to forms of inequality; culpability, feelings of being responsible for their family member’s serious mental illness; and devaluation, emotional reactions and responses to feeling less valuable, or that their value has been depreciated). However, after analysing the psychometric properties of the scale in terms of internal consistency and exploratory factor analysis, the results were very different. In this sense, the authors showed that the exploratory factor analysis of the scale reflected a monofactorial model to the detriment of the 5-factor model theoretically proposed by the authors (for more information, see the study by Morris, et al. [19]). This scale has been validated in the Spanish context [20], showing that the psychometric properties were adequate for both the 5-factor model and the single-factor model through a higher-order structural equation model. The relationships between the factors in the 5-factor model were positive, showing reciprocity between the factors. In addition, the scale showed adequate reliability indices above 0.80.

Thus, the present study aims to adapt and show evidence of the validity of the Self-Stigma in Relatives of People with Mental Illness Scale to the Spanish context referred to autism. In order to analyse its reliability and validity, several analyses will be carried out (e.g., confirmatory factor analysis, Cronbach’s alpha, McDonald’s omega, descriptive statistics and multi-group analysis to analyse gender invariance). It is expected that the scale will show good fit indices, as in the original version, and that it will be invariant with respect to gender.

## 2. Method

### 2.1. Participants

In order to carry out this study, the collaboration of 287 progenitors with children with autism (149 women and 138 men) was needed. The mean age of the participants was 45.68 years old (SD = 8.47), between 27 and 63 years old, belonging to the province of Almeria and the community of Murcia. A non-probabilistic incidental sampling method was used, since all participants were first-degree relatives of people diagnosed with autism spectrum disorder.

### 2.2. Measurements

To measure the self-stigma perceived by relatives with a son or daughter with autism spectrum disorder, the Self-Stigma in Relatives of people with Mental Illness (SSRMI) by Morris, et al. [19] was validated and adapted. For this purpose, items referring to severe mental disorder were initially changed to autism spectrum disorder. Thus, the heading of the questionnaire has the following sentence:

*“Las siguientes preguntas hacen mención a cómo se siente actualmente acerca de la enfermedad de su hijo. Aunque usamos el término "enfermedad", piense en esto de cualquier manera que se sienta más cómodo. Marque si está totalmente en desacuerdo, en desacuerdo, se siente neutral, de acuerdo o muy de acuerdo con las siguientes afirmaciones.”*

The 30 items that make up the scale (items 1, 11, 18, 26, 27 and 28 are reverse coded) are divided into five factors: devaluation, guilt, separation, discrimination and stereotyping.

Participants were asked to indicate their response using a Likert scale from 1 (strongly disagree) to 5 (strongly agree). 

Morris, et al. [19] and Trigueros, et al. [20] propose a short version of the SSRMI consisting of ten items, corresponding to items 6, 13, 14, 15, 16, 21, 23, 24, 25 and 30.

### 2.3. Procedure

To validate the scale, some of the items referring to severe mental disorder were modified to adapt it to the autism spectrum context. Once the final version of the scale was obtained, it was analysed by three experts in clinical psychology, external to the research group, with extensive experience in hospital centres, early childhood care and research experience, ensuring that the items obtained were well designed to measure the construct to be measured, without losing the original meaning [21].

Before starting the collection of information, the favourable approval of the Bioethics Committee of the University of Almeria in Human Research (Ref. UALBIO 2019/014) was requested. Once this approval was obtained, various medical centres and associations in Andalusia and Murcia were contacted to request their collaboration, initially informing them of the objective of the research. Family members were asked to sign an informed consent form to participate in the study. Before administering the scale to all participants, it was completed by a small group of people to ensure correct understanding of all items. The questionnaire was completed by the participants under the supervision of the principal investigator, who explained and resolved any doubts that arose during the completion of the questionnaire. The application of the questionnaire was carried out in the clinical services. The estimated time to complete the questionnaire was around 15 min.

### 2.4. Data Analysis

In order to test the factorial structure and reliability of the scale in the Spanish context, the psychometric properties of the questionnaire were analysed. First, a confirmatory factor analysis (CFA) was carried out to test the factor structure of the 30-item questionnaire, a second confirmatory factor analysis of higher order and a third confirmatory factor analysis of the short 10-item version of the one-factor scale. Secondly, multigroup analyses were conducted to analyse invariance with respect to gender for both the 30-item scale and the 10-item scale. Next, descriptive statistical analyses were conducted and the reliability of the instrument was tested (Cronbach’s alpha and McDonald’s omega). The AMOS 25.0 and SPSS 22.0 statistical packages were used for data analysis.

The bootstrapping procedure (6000 interactions), together with the maximum likelihood method, was used for the CFA as the Mardia coefficient showed a high score (328.75). Furthermore, the estimators were considered robust as they were not affected by non-normality [22]. The fit indices taken into account are detailed below [23]: *χ*^2^/*df* with values between 2 and 3; the incremental indices (CFI, Comparative Fit Index; IFI, Incremental Fit Index; and TLI, Tucker Lewis Index) with values above 0.95, RMSEA (Root Mean Square Error of Approximation) with values below 0.08, plus its 90% confidence interval (CI), and SRMSR (Standardized Root Mean Square Residual) with values below 0.06.

## 3. Results

### 3.1. Confirmatory Factorial Analysis

**Figure 1:**
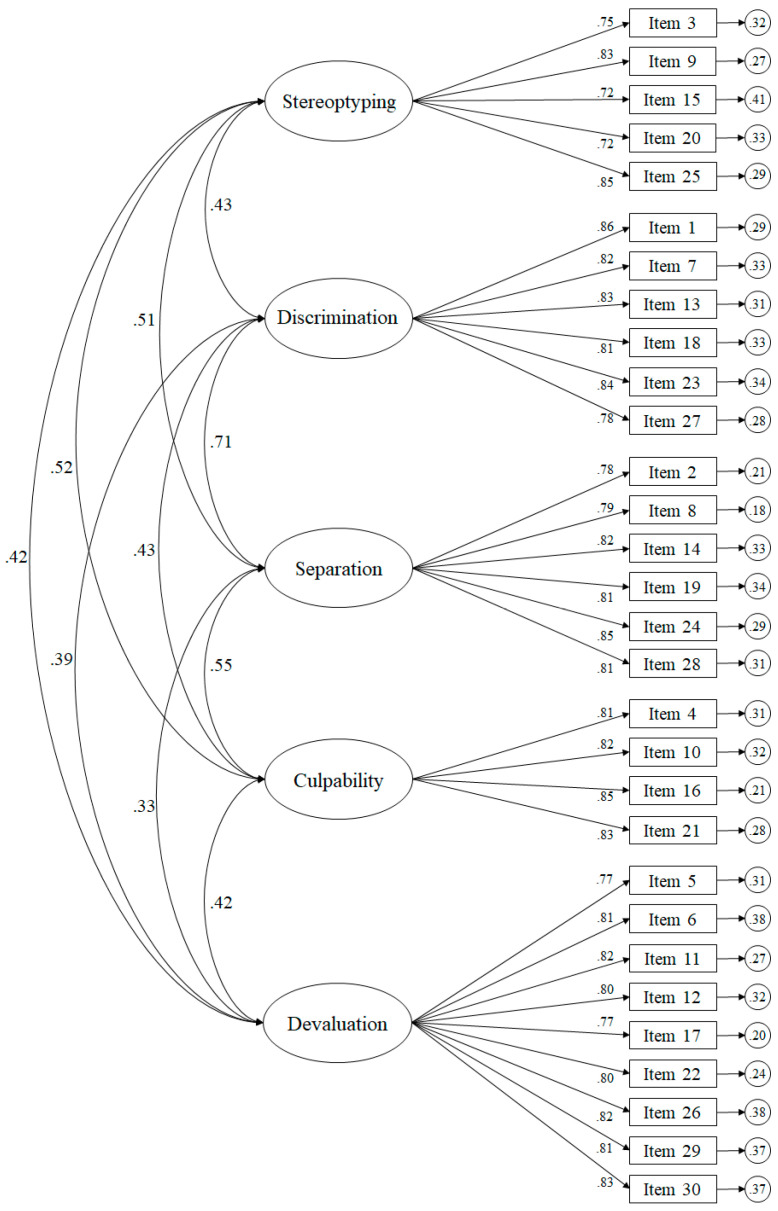
Confirmatory factor analysis of the 30-item scale in the Spanish context. The ellipses represent the factors and the rectangles represent the different items. The residual variances are shown in the small circles.

The model tested (Figure 1) through CFA revealed appropriate fit indices for the 30-item model: *χ*^2^ (395. N = 316) = 987.23, *p* < 0.001; *χ*^2^/*df* = 2.50; CFI = 0.96; TLI = 0.96; IFI = 0.96; RMSEA = 0.059 (90% CI = 0.055–0.068); SRMSR = 0.049. The standardised regression weights ranged from 0.72 to 0.86 and were statistically significant (*p* < 0.001). The correlation between the factors ranged from 0.33 to 0.71 and was statistically significant (*p* < 0.001). In relation to the 10-item model, the following were equally acceptable: *χ*^2^ (35. N = 304) = 126.45, *p* < 0.001; *χ*^2^/*df* = 2.61; CFI = 0.94; TLI = 0.94; IFI = 0.94; RMSEA = 0.072 (90% CI = 0.070–0.079); SRMSR = 0.049. The standardised regression weights were around 0.52 and 0.87.

Finally, the higher order model revealed appropriate fit indices: *χ*^2^ (400. N = 304) = 951.37, *p* < 0.001; *χ*^2^/*df* = 2.37; CFI = 0.94; TLI = 94; IFI = 0.94; RMSEA = 0.066 (90% CI = 0.063–0.072); SRMSR = 0.057. There was a correlation between the higher order factor, called Self-stigma, with respect to separation of 0.75, discrimination of 0.64, stereotyping of 0.67, guilt of 0.77 and devaluation of 0.51.

### 3.2. Gender Invariance Analysis

As shown in Table 1, no significant differences were found between model 1 (unrestricted model) and model 2 (model with invariance in measurement weights), however, there were significant differences with model 3 (model with invariant structural covariances) and model 4 (model with invariant measurement residuals). The absence of significant differences between model 1 and model 2 is a minimum criterion for accepting that the model structure is gender invariant [24]. Furthermore, in Table 1, ∆CFI of model 1 vs. model 3, for example, would give a 0.01 and a ∆RMSEA of 0.006, which can be considered as a proof of invariance [25].

Similarly, the results also support gender invariance for the 10-item model, as no significant differences were found between model 1 (unrestricted model), model 2 (measurement weights invariance model) and model 3 (structural weights invariant model). However, they did show significant differences between model 1 and model 4 (invariant structural covariance model).

### 3.3. Descriptive Statistics, Correlation and Reliability Analysis

Table 2 shows that the correlation between the five factors is positive and significant, which highlights the clear reciprocity between the factors. Likewise, in order to obtain evidence of the reliability of the scale, two reliability analyses were carried out where the scores were satisfactory, above 0.80.

Finally, the correlation between the higher order factor from the 30-item scale correlated positively (*β* = 0.54; *p* < 0.001) with the 10-item unifactor scale.

## 4. Discussion

The aim of the present study was to test the factor structure of the Self-Stigma Scale in Relatives of People with Autism (SSRA), analysing its psychometric properties. For this purpose, three confirmatory factor analyses, a gender invariance analysis, an internal consistency analysis and descriptive statistics were conducted. The results of the analyses supported the SSRA as a valid, reliable and stable instrument for assessing relatives’ self-stigma.

The AFC of the SSRA revealed that the factor structure of the questionnaire consisted of five factors. On the other hand, the higher-order model also showed adequate psychometric properties, so that if researchers decided to conduct a study using the scale, they could simplify it by grouping the five factors into one. Furthermore, as with the original scale, the factor structure of the short version of the instrument was analysed, showing adequate psychometric properties. These results are similar to those achieved in the study by Trigueros et al. [20], where it was shown that the factor structure of the questionnaire consisted of five factors. However, they are contradictory to the initial scale of Morris, et al. [19], where the authors created a scale composed of five theoretical factors, but after psychometric analyses the scale showed a unifactorial structure. In this sense, the construction of a scale is a continuous process where, through successive studies, the scale is refined. Thus, a study by Mills, et al. [26] using the SSRMI version showed that those family members who had high levels of stereotyping, discrimination, separation, guilt and devaluation showed a greater predisposition towards depression; therefore, the researchers used the scale in a multifactorial way. Similarly, a study by Trigueros, et al. [27] showed how relatives’ self-stigma positively predicted burnout. This study used a higher-order model to measure self-stigma.

On the other hand, the results reflected that the scale showed adequate levels of reliability, as each of the items, as well as the overall factor, showed a score above 0.80. Furthermore, the results of the temporal stability analysis through the R-test showed that the scale items are understood in a similar way after two weeks, which is enough time for people’s opinions not to change due to their experiences. Furthermore, the questionnaire is understood in the same way irrespective of gender, so future studies could carry out comparative studies between men and women.

Despite the positive results of the study, there are a number of limitations that must be taken into account. In this sense, the sample selection has been non-probabilistic and incidental, so that all populations may not be reflected in the study. On the other hand, this study has not considered other factors related to children with ASD (e.g., type, characteristics, place of residence, family economic status, etc.) that may influence the generalisability of the results. Furthermore, the questionnaire is based on self-reported measures. Finally, future studies should analyse the factor structure through its relationship with other determinants of social self-stigma. On the other hand, future studies should also analyse the differences between fathers and mothers, and analyse the effects of self-stigma on parenting styles.

## 5. Conclusions

Despite the limitations, the results have supported the psychometric properties of the study as an instrument that shows evidence of validity and reliability, which may be useful to the scientific community for research in a new field of study. Furthermore, the use of the scale may be useful for social and clinical psychologists to facilitate the design of treatments that can minimise or eliminate the impact of self-stigma in these situations [28]. In addition, a short version of the instrument is available, which will allow practitioners to obtain information in a very short space of time.

## Figures and Tables

**Table 1:** Gender Invariance Analysis.

Full Version (30 items)										
**Models**	***χ*^2^**	***df***	***χ*^2^/*df***	**Δ*χ*^2^**	**Δ*df***	**CFI**	**TLI**	**IFI**	**RMSEA (IC 90%)**	**SRMSR**
Model 1	1761.71	790	2.23	-	-	0.97	0.97	0.97	0.052 (0.049–0.060)	0.042
Model 2	1882.66	815	2.31	31.26	25	0.97	0.97	0.97	0.054 (0.050–0.060)	0.043
Model 3	2044.91	845	2.42	90.66 **	55	0.96	0.96	0.96	0.058 (0.051–0.062)	0.046
Model 4	2218.81	860	2.58	134.67 ***	70	0.95	0.95	0.95	0.056 (0.054–0.061)	0.047
Short Version (10 items)										
**Models**	***χ*^2^**	***df***	***χ*^2^/*df***	**Δ*χ*^2^**	**Δ*df***	**CFI**	**TLI**	**IFI**	**RMSEA (IC 90%)**	**SRMSR**
Model 1	188.82	79	2.39	-	-	0.95	0.95	0.95	0.059 (0.055–0.063)	0.053
Model 2	231.41	89	2.60	14.29	9	0.95	0.95	0.95	0.060 (0.055–0.068)	0.052
Model 3	241.20	90	2.68	26.44	20	0.94	0.94	0.94	0.063 (0.065–0.070)	0.058
Model 4	293.88	100	2.94	57.33 **	30	0.94	0.94	0.94	0.062 (0.059–0.071)	0.054

*** *p* < 0.001; ** *p* < 0.01.

**Table 2:** Descriptive Statistics, Cronbach’s Alpha and Bivariate Correlations.

Factors	*M*	*SD*	α	ω	1	2	3	4	5
1. Stereotyping	1.81	0.80	0.80	0.83		0.66 ***	0.53 **	0.64 ***	0.54 ***
2. Culpability	2.12	1.33	0.83	0.85			0.31 **	0.45 **	0.61 ***
3. Devaluation	1.67	0.80	0.85	0.87				0.43 ***	0.63 ***
4. Discrimination	1.23	1.34	0.81	0.82					0.40 ***
5. Separation	2.11	1.05	0.79	0.83					

*** *p* < 0.001; ** *p* < 0.01.

## Data Availability

The data presented in this study are available on request from the corresponding author.

## Appendix A

This scale was validated in Spanish, not in English.

1. Me sentiría cómodo diciéndole a mis amistades que mi hijo/a tiene TEA.
  I would feel comfortable telling my friends that my child has ASD.
2. Necesito esconder el trastorno de mi hijo/a.
  I need to hide my child’s disorder.
3. El TEA de hijo/a se refleja negativamente en mí.
  My child’s ASD reflects negatively on me.
4. Me siento culpable porque mi hijo/a tenga TEA.
  I feel guilty about my child having ASD.
5. El TEA de mi hijo/a, me hace sentir incómodo cuando estamos en situaciones sociales.
  My child’s ASD makes me feel uncomfortable when we are in social situations.
6. Me siento abochornado por tener un hijo/a con TEA.
  I feel embarrassed about having a child with ASD.
7. No puedo vivir mi vida de la manera que quiero porque tengo un hijo/a con TEA.
  I can’t live my life the way I want to because I have a child with ASD.
8. Tengo que ser selectivo con quien le cuente que tengo un hijo/a con TEA.
  I have to be selective about who I tell that I have a child with ASD.
9. Las personas con TEA no deberían de tener hijos.
  People with ASD should not have children.
10. 1Me siento responsable de causar el trastorno de mi hijo/a.
  I feel responsible for causing my child’s disorder.
11. Mi vida es más plena porque tengo un hijo/a con TEA.
  My life is fuller because I have a child with ASD.
12. Me siento avergonzado por tener un hijo/a con TEA.
  I feel ashamed that I have a child with ASD.
13. Tener un hijo/a con TEA me ha hecho preocuparme más por mí.
  Having a child with ASD has made me care more about myself.
14. La gente no quiere hablar conmigo debido al TEA de mi hijo/a.
  People don’t want to talk to me because of my child’s ASD.
15. Me preocupa ser etiquetado como alguien que tiene un hijo/a con TEA.
  I worry about being labelled as someone who has a child with ASD.
16. La gente me culpa de la enfermedad de mi hijo/a.
  People blame me for my child’s condition.
17. Mi identidad se ha visto negativamente afectada por la trastorno de mi hijo/a.
  My identity has been negatively affected by my child’s condition.
18. Tengo la esperanza de que algún día el trastorno del espectro autista serán tratadas con carácter normalizado.
  I am hopeful that one day the autistic spectrum disorder will be treated as normalised.
19. Me siento fuera de lugar en el mundo porque tengo un hijo/a con TEA.
  I feel out of place in the world because I have a child with ASD.
20. Sigo buscando señales de que mi familiar no tiene realmente TEA.
  I keep looking for signs that my family member does not really have ASD.
21. Me culpo por el trastorno de mi hijo/a.
  I blame myself for my child’s disorder.
22. Cuando mi hijo/a con TEA es juzgado, me siento juzgado también.
  When my child with ASD is judged, I feel judged too.
23. Me siento discriminado/a porque tengo un hijo/a con TEA.
  I feel discriminated against because I have a child with an ASD.
24. Me siento aislado/a porque tengo un hijo/a con TEA.
  I feel isolated because I have a child with an ASD.
25. Minimizo la gravedad del TEA de mi hijo/a cuando lo describo a las personas.
  I minimise the severity of my child’s ASD when I describe it to people.
26. Soy una persona más fuerte porque tengo un hijo/a con TEA.
  I am a stronger person because I have a child with ASD.
27. Los profesionales que trabajan con mi hijo/a valoran mi conocimiento acerca del TEA.
  The professionals who work with my child value my knowledge about ASD.
28. Puedo hablar abiertamente sobre TEA con otros miembros de mi familia.
  I am able to talk openly about ASD with other members of my family.
29. Me siento devastado/a de que mi hijo/a tenga TEA.
  I feel devastated that my child has ASD.
30. Mi autoestima se ha visto deteriorada debido al trastorno de mi hijo/a.
  My self-esteem has deteriorated because of my child’s disorder.

## References

[1] López S., Rivas R., Taboada E. (2009). Revisiones sobre el autismo. Rev. Latinoam. De Psicol..

[2] Mottron L., Bzdok D. (2020). Autism spectrum heterogeneity: Fact or artifact?. Mol. Psychiatry.

[3] Grove J., Ripke S., Als T.D., Mattheisen M., Walters R.K., Won H., Pallesen J., Agerbo E., Andreassen O.A., Anney R. (2019). Identification of common genetic risk variants for autism spectrum disorder. Nat. Genet..

[4] Loomes R., Hull L., Mandy W.P.L. (2017). What is the male-to-female ratio in autism spectrum disorder? A systematic review and meta-analysis. J. Am. Acad. Child Adolesc. Psychiatry.

[5] van‘t Hof M., van Berckelaer-Onnes I., Deen M., Neukerk M.C., Bannink R., Daniels A.M., Hoek H.W., Ester W.A. (2020). Novel insights into autism knowledge and stigmatizing attitudes toward mental illness in Dutch youth and family center physicians. Community Ment. Health J..

[6] Carbajosa A.B., Pérez F.B., Bertina A., Quintana Y.C., Sánchez M.B.M., Galán S.P. (2018). La dinámica estigmatizante: Generación y mantenimiento del estigma y el autoestigma asociado al trastorno mental en la vida cotidiana. Clínica Contemp..

[7] Corrigan P.W., Nieweglowski K. (2019). How does familiarity impact the stigma of mental illness?. Clin. Psychol. Rev..

[8] Midence K., O’neill M. (1999). The experience of parents in the diagnosis of autism: A pilot study. Autism.

[9] Woodgate R.L., Ateah C., Secco L. (2008). Living in a world of our own: The experience of parents who have a child with autism. Qual. Health Res..

[10] Plotkin S., Gerber J.S., Offit P.A. (2009). Vaccines and autism: A tale of shifting hypotheses. Clin. Infect. Dis..

[11] Kelly A.B., Garnett M.S., Attwood T., Peterson C. (2008). Autism spectrum symptomatology in children: The impact of family and peer relationships. J. Abnorm. Child Psychol..

[12] Gross L. (2009). A Broken Trust: Lessons from the Vaccine–Autism Wars: Researchers long ago rejected the theory that vaccines cause autism, yet many parents don’t believe them. Can scientists bridge the gap between evidence and doubt?. PLoS Biology.

[13] Ludlow A., Skelly C., Rohleder P. (2012). Challenges faced by parents of children diagnosed with autism spectrum disorder. J. Health Psychol..

[14] Tárraga-Mínguez R., Gómez-Marí I., Sanz-Cervera P. (2020). What Motivates Internet Users to Search for Asperger Syndrome and Autism on Google?. Int. J. Environ. Res. Public Health.

[15] Zhang L., Haller B. (2013). Consuming image: How mass media impact the identity of people with disabilities. Commun. Q..

[16] Bergold S., Hastall M.R., Steinmayr R. (2021). Do Mass Media Shape Stereotypes About Intellectually Gifted Individuals? Two Experiments on Stigmatization Effects from Biased Newspaper Reports. Gift. Child Q..

[17] Alshaigi K., Albraheem R., Alsaleem K., Zakaria M., Jobeir A., Aldhalaan H. (2020). Stigmatization among parents of autism spectrum disorder children in Riyadh, Saudi Arabia. Int. J. Pediatrics Adolesc. Med..

[18] Chan K.K.S., Lam C.B. (2018). Self-stigma among parents of children with autism spectrum disorder. Res. Autism Spectr. Disord..

[19] Morris E., Hippman C., Murray G., Michalak E.E., Boyd J.E., Livingston J., Inglis A., Carrion P., Austin J. (2018). Self-stigma in relatives of people with mental illness scale: Development and validation. Br. J. Psychiatry.

[20] Trigueros R., Aguilar-Parra J.M., Cangas A.J., Ortiz L., Navarro N. (2019). Adaptation and validation to the spanish context of the scale of self-stigma in relatives of people with mental illness. Ann. Psychol..

[21] Lynn M.R. (1986). Determination and quantification of content validity. Nurs. Res..

[22] Byrne B.M. (2001). Structural equation modeling with AMOS, EQS, and LISREL: Comparative approaches to testing for the factorial validity of a measuring instrument. Int. J. Test..

[23] Hair J.F., Black W.C., Babin B.J., Anderson R.E., Tatham R.L. (2006). Multivariate Data Analysis.

[24] Byrne B.M., Shavelson R.J., Muthén B. (1989). Testing for the equivalence of factor covariance and mean structures: The issue of partial measurement invariance. Psychol. Bull..

[25] Cheung G.W., Rensvold R.B. (2002). Evaluating goodness-of-fit indexes for testing measurement invariance. Struct. Equ. Modeling.

[26] Mills L., Meiser B., Ahmad R., Schofield P.R., Peate M., Levitan C., Mills L., Meiser B., Ahmad R., Schofield P.R. (2019). A cluster randomized controlled trial of an online psychoeducational intervention for people with a family history of depression. BMC Psychiatry.

[27] Trigueros R., Navarro N., Cangas A.J., Mercader I., Aguilar-Parra J.M., González-Santos J., González-Bernal J.J., Soto-Cámara R. (2020). The Protective Role of Emotional Intelligence in Self-Stigma and Emotional Exhaustion of Family Members of People with Mental Disorders. Sustainability.

[28] Navarro-Bayón D., García-Heras S., Carrasco O., Casas A. (2008). Calidad de vida, apoyo social y deterioro en una muestra de personas con trastorno mental grave. Psychosoc. Interv..

